# Complement activation contributes to subretinal fibrosis through the induction of epithelial-to-mesenchymal transition (EMT) in retinal pigment epithelial cells

**DOI:** 10.1186/s12974-022-02546-3

**Published:** 2022-07-14

**Authors:** María Llorián-Salvador, Eimear M. Byrne, Manon Szczepan, Karis Little, Mei Chen, Heping Xu

**Affiliations:** 1 The Wellcome-Wolfson Institute for Experimental Medicine, School of Medicine, Dentistry & Biomedical Science, Queen’s University Belfast, 97 Lisburn Road, Belfast, BT9 7BL Northern Ireland UK; 2 Present Address: Vall d´Hebron Research Institute (VHIR), Universitat Autonòma de Barcelona, 08035 Barcelona, Spain; 3 Present Address: Centre for Genomic Regulation (CRG), The Barcelona Institute of Science and Technology, 08003 Barcelona, Spain

**Keywords:** Age-related macular degeneration, Macular fibrosis, Inflammation, Complement system, Retinal pigment epithelial cell, C5a, C3a, Subretinal fibrosis, Epithelial-to-mesenchymal transition

## Abstract

**Background:**

We previously reported higher plasma levels of complement fragments C3a and C5a in neovascular Age-related Macular Degeneration (nAMD) patients with macular fibrosis. This study aimed to understand whether complement activation contributes to the development of macular fibrosis and the underlying mechanisms involved.

**Methods:**

Complement activation was blocked using a C5 neutralizing antibody (BB5.1) in C57BL/6J mice after induction of subretinal fibrosis using the two-stage laser protocol. Fibrotic lesions were examined 10 days after the 2nd laser through fundus examination and immunohistochemistry. The expression of C5aR in fibrotic lesions and retinal pigment epithelial (RPE) cultures were examined by confocal microscopy. Primary murine RPE cells were treated with C3a or C5a (10–100 ng/mL) or TGF-β2 (10 ng/mL). Epithelial-to-mesenchymal transition (EMT) was assessed through various readouts. The expression of E-cadherin, vimentin, fibronectin, α-SMA, Slug, ERK/AKT and pSMAD2/3 were determined by Western blot and immunocytochemistry. Collagen contraction and wound-healing assays were used as functional readouts of EMT. The production of IL-6, TGF-β1, TGF-β2 and VEGF by RPE cells were determined by ELISA. PMX53 was used to block C5aR in RPE cultures and in vivo in mice with subretinal fibrosis.

**Results:**

Extensive C5b-9 deposition was detected at the site of subretinal fibrosis. BB5.1 treatment completely abrogated complement activation and significantly reduced subretinal fibrosis. C5aR was detected in RPE and infiltrating MHC-II^+^ cells in subretinal fibrosis. In vitro, RPE cells constitutively express C5/C5a and C5aR, and their expression was increased by TGF-β2 treatment. C5a but not C3a increased fibronectin, α-SMA, vimentin and Slug expression, and decreased E-cadherin expression in RPE cells. C5a treatment also increased the contractility and migration of RPE cells and enhanced the production of VEGF and TGF-β1/2. C5a treatment induced pSmad2/3 and pERK1/2 expression in RPE cells and this was blocked by PMX53. PMX53 treatment significantly reduced sodium fluorescein leakage in the subretinal fibrosis model, while collagen-I^+^ lesions only mildly reduced.

**Conclusions:**

Complement activation is critically involved in the development of subretinal fibrosis, partially through C5a–C5aR-mediated EMT in RPE cells. Targeting complement activation rather than C5a may be a novel approach for the management of macular fibrosis.

**Supplementary Information:**

The online version contains supplementary material available at 10.1186/s12974-022-02546-3.

## Background

Age-related macular degeneration (AMD) is the leading cause of blindness in developed countries. It has been estimated that 288 million people will be affected by this condition by 2040 [1, 2]. Neovascular AMD (nAMD) causes severe vision loss and is characterized by the macular invasion of abnormal blood vessels from the choroid (e.g., choroidal neovascularization, CNV, or polypoidal choroidal vasculopathy, PCV) or the retina (i.e. retinal angiomatous proliferation, RAP). These new vessels can subsequently lead to the formation of subretinal fibrotic plaques in the macula, known as macular fibrosis [3–5]. Anti-VEGF therapy is the mainstay of nAMD treatment [6]. Although anti-VEGF therapy can stabilize and even improve visual function in nAMD, nearly half of patients suffer from poor prognosis, largely due to the development of macular fibrosis [5]. Currently, there are no medications to prevent or treat macular fibrosis. Therefore, novel strategies based on a better understanding of disease pathogenesis are urgently needed.

Macular fibrosis originates from new blood vessels and is a fibro-vascular membrane [7]. The conversion of the diseased blood vessels into a fibro-vascular lesion is due to excess deposition of extracellular matrix (ECM) from activated fibroblasts (myofibroblasts). The retina, including the macula, is absent of fibroblasts. Evidence suggests that myofibroblasts can be recruited from the choroid and blood circulation. They may also be transdifferentiated from other cells such as Müller cells, endothelial cells, retinal pigment epithelial (RPE) cells and infiltrating macrophages [7, 8]. For example, RPE cells can transdifferentiate into myofibroblasts through epithelial-to-mesenchymal transition (EMT) and this process is believed to play a pivotal role in AMD [3, 5, 7, 9, 10]. The characteristics of EMT in RPE cells include the loss of epithelial features such as cell-to-cell contact, decreased expression of adherence and tight junction proteins. Additionally, the gain of mesenchymal features such as increased cell migration and invasiveness, higher levels of contractility and upregulation of ECM proteins such as collagen type I (Col-I) and fibronectin (FN) and α-smooth muscle actin (α-SMA). The molecular cues governing the transdifferentiation of myofibroblasts from other cells in the context of macular fibrosis remain poorly defined, although sustained inflammation is believed to be the main driver of pathogenic fibrosis.

The complement system is involved in chronic inflammation of many degenerative diseases, including AMD [11, 12]. In recent years, emerging evidence suggests that the complement system also plays a critical role in organ fibrosis such as the lung [13, 14], kidney [15–19] and liver [20]. The complement fragments C3a and C5a are cleavage products of C3 and C5 during complement activation and are involved in diverse immune responses. C3a and C5a can bind their cognate receptors C3aR and C5aR initiating pro-inflammatory, pro-angiogenic and pro-fibrotic responses in both immune and tissue cells. Therefore, they are critically involved in tissue regeneration, remodelling and fibrosis. We previously reported higher plasma levels of C3a and C5a in nAMD patients with subretinal fibrosis [21]. We further found that C3a but not C5a could induce macrophage-to-myofibroblasts transition [8]. This study aimed to understand whether C3a and C5a could induce EMT in RPE cells in the context of subretinal fibrosis and whether blocking complement activation could prevent or reduce retinal fibrosis.

## Materials and methods

### Human eyes

Human eye samples with nAMD were obtained from the San Diego Eye Bank. This study was carried out within the parameters of the Declaration of Helsinki, and tissues were stored in accordance with the UK Human Tissue Act (2004). The research was approved by the Ethical Review Boards of Queen’s University Belfast. The eyes were maintained in formalin. Upon arrival, the eyes were dissected and embedded in paraffin and sectioned at 6 µm thickness.

### Animals

C57BL/6J mice aged between 2 and 4 months were used in this study. All animals were housed and bred in a standard pathogen-free experimental facility and exposed to a 12-h light/dark cycle with free access to food and water. All procedures were conducted under the regulation of the UK Home Office Animals (Scientific Procedures) Act 1986. This study was approved by the Animal Welfare and Ethical Review body (AWERB) of Queen's University Belfast and conducted in compliance with the Association for Research in Vision & Ophthalmology Statement for the Use of Animals in Ophthalmology and Vision Research.

### Two-stage laser-induced subretinal fibrosis

Subretinal fibrosis was induced using a two-stage laser protocol previously described by our group [8, 22]. Briefly, CNV was induced by using the laser photocoagulator (HGM Medical Laser System Inc. Salt Lake City, USA). The settings for the laser were as follows: laser power, 250 mv; duration, 0.1 s; and spot size, 100 μm. Four laser spots were delivered per eye. Seven days later, a second laser burn was applied to each CNV lesion using the same laser configuration.

### Inhibition of C5 or C5aR in vivo in subretinal fibrosis

To inhibit complement activation, mice were injected intraperitoneally with 250 µg per animal of C5 blocking antibody, BB5.1 (Cat. HM1073, Hycult, Uden, Netherland) 1 h before the second laser injury (day 0). A second injection was performed 5 days later (125 µg/animal). The doses were chosen based on a previous study of this antibody in experimental autoimmune uveitis [23]. Control mice were injected with the same amount of isotype control (mouse IgG, Cat. MAB002, R&D Systems, Minneapolis, MN). Peripheral blood was collected before the first laser, 24 h after each injection (day 1 and 6) and at the endpoint (day 10) and the serum was isolated and used for the complement activation assays.

To block C5aR, mice were subcutaneously injected daily with the C5aR antagonist PMX53 (1 mg/kg, Cat. 5473, Tocris, Bio-techne, Minneapolis, MN) starting 1 h after the second laser (day 0) until the end point. Control mice were injected with the same volume of vehicle (saline).

### Fundus fluorescence angiography

Fundus images and fundus fluorescein angiography (FFA) were conducted on day 10 post second laser, using the Micron IV system and the Discover 2.2 Programme (Phoenix Technology Group, Pleasanton, CA). FFA was carried out 5 min after intra-peritoneal injection of 100 μL of 10% sodium fluorescein (Sigma-Aldrich, Gillingham, UK, Cat. F6377). Exposure level was kept consistent between animals. The area of fluorescein leakage from each lesion were analysed using ImageJ (NIH, Bethesda, MD) by two independent researchers in a masked fashion. Following retinal fundus (Micron IV) examination, mice were killed by CO_2_ and eyes were collected and fixed in 2% paraformaldehyde for 2 h (Sigma-Aldrich, Cat. 158127) and processed for RPE/choroidal flatmount staining.

### Complement activity assay

Classic complement system activation in serum from mice treated with BB5.1 C5 antibody or control mouse IgG was determined using the Hycult Mouse Classical Complement Pathway assay (HIT420, Hycult Biotech, Uden, Netherlands) following manufacturer’s instructions. Besides negative and positive controls provided in the kit, a reference of total complement activity was created by stimulating a pool of the serum samples from the control animals with 2 µg/mL LPS for 2 h 37 °C. Unstimulated plasma was considered as reference negative control. Complement activity (%) of each sample was calculated as follows: (sample – reference negative control)/(total complement activation − reference negative control) × 100.

### Primary culture of RPE cells

Primary mouse RPE cells were cultured using the protocol previously described [24, 25]. Briefly, eyes were collected from 2- to 3-month-old C57BL/6J mice. Anterior segment of the eye (cornea, lens, iris and ciliary body) was removed. The retina was carefully peeled off of the RPE/choroidal eyecup. The RPE/choroidal eyecups were incubated with pre-heated 0.05% trypsin (Gibco, Cat. 10779413) for 45 min at 37 °C. The RPE cells were flushed from the eyecups and cultured in Dulbecco’s modified Eagle medium: nutrient mixture F-12 (DMEM/F12, Cat. 11320033, Gibco, Waltham, MA) supplemented with 15% FCS (Gibco™, Cat. 10270106) and 1% penicillin–streptomycin (Gibco, Cat. 15140122). The phenotype of RPE cells was confirmed by RPE65 staining. Cells from passages 3–5 were used in the study. For experiments, media was changed to lower serum (1% FCS) when fully confluent to facilitate cell quiescence. 24 h later, the different treatments (C3a, C5a or TGF-β2, concentrations ranging from 10 to 100 ng/mL [8, 16] or 50 nM of PMX53, all dissolved in PBS) were added in DMEM-F12 supplemented with 1% FCS for different lengths of time. The sources of these recombinant proteins are detailed in Table 1.

### Immunostaining

Cells were fixed in 2% paraformaldehyde for 20 min, rinsed in PBS, and blocked with 10% BSA (Sigma-Aldrich) and permeabilized with 0.1% Triton X-100 (Sigma-Aldrich). Cells were then incubated overnight (4 °C) with primary antibodies (Table 1) diluted in PBS. Following incubation, cells were incubated with fluorophore-conjugated secondary antibodies (Table 2) at room temperature for 1 h. Cells were counterstained using DAPI-Vectashield (Vector Labs, Burlingame, CA) and examined under Leica DMi8 epifluorescence microscope.

**Table 1:** Recombinant proteins and primary antibodies used in the study

Recombinants/molecules		
Product name	Cat. No.	Company
Recombinant Mouse Complement C3a, CF	8085-C3	R&D Systems
Recombinant Mouse Complement C5a	2150-C5-025	R&D Systems
Recombinant Mouse TGF-β2	7346-B2-005	R&D Systems
PMX 53 (C5a receptor antagonist)	Cat. No. 5473	Tocris-Biotechne

Antibodies			
Product name	Cat. No.	Company	Host
Fibronectin	ab2413	Abcam	Rabbit
E Cadherin antibody	orb213706	Biorbyt	Rabbit
α-SMA (-Cy3 conjugated)	C6198	Sigma-Aldrich	Mouse
Rabbit anti-α-SMA	ab5694	Abcam	Rabbit
C5b-9	ab65811	Abcam	Rabbit
C5aR	ab59390	Abcam	Rabbit
C5aR	ab117579	Abcam	Rat
C5R1 Biotin	ab54378-100	Abcam	Biotin
C5a antibody (FITC)	orb360860	Biorbyt	Rat
C5a antibody	MAB21501-100	R&D Systems	Goat
Collagen 1 Rabbit pAb	ab34710	Abcam	Rabbit
Gt X Collagen Type I	AB758	CHEMICON	Goat
Vimentin polyclonal antibody	Orb304659	Biorbyt	Rabbit
Slug antibody	PSI-3957	ProSci	Rabbit
MHC Class II (I-A/I-E) Monoclonal Antibody	14–5321-82	eBioscience	Rat
GAPDH	G8795	Sigma	Mouse
AKT/MAPK Pathway Antibody Cocktail	ab151279	Abcam	Rabbit
pSmad2/3	8828	Cell Signaling Technologies	Rabbit

**Table 2:** Secondary antibodies and ELISA kits used in the study

Product name	Cat. No.	Company
*Secondary antibodies*		
Alexa Fluor® 594 AffiniPure Donkey Anti-Rabbit IgG (H + L)	711–585-152	Jackson Immunoresearch
Alexa Fluor® 594-AffiniPure Donkey Anti-Goat IgG (H + L)	705–585-147	Jackson Immunoresearch
Alexa Fluor® 488-AffiniPure Donkey Anti-Goat IgG (H + L)	705–545-147	Jackson Immunoresearch
Alexa Fluor® 488-AffiniPure Donkey Anti-Rabbit IgG (H + L)	711–545-152	Jackson Immunoresearch
Alexa Fluor® 488-AffiniPure Fab Donkey Anti-Rat IgG (H + L)	712–547-003	Jackson Immunoresearch
Goat Anti-Rabbit IgG H&L (HRP)	ab6721	Abcam
Rabbit Anti-Mouse IgG H&L (HRP)	ab6728	Abcam
Rabbit Anti-Goat IgG H&L (HRP)	ab6741	Abcam
*ELISA kits*		
IL-6	BMS603-2	Thermo Fisher Scientific
TNF-α	BMS607-3	Thermo Fisher Scientific
Mouse TGF-beta 2 DuoSet ELISA	DY7346-05	R&D
Mouse VEGF DuoSet	DY493-05	R&D
Mouse Complement Component C5a DuoSet ELISA	DY2150	R&D
Mouse TGF-beta 1 DuoSet ELISA	DY1679-05	R&D
C5 ELISA	ORB565566	Biorbyt

RPE flatmounts were stained as previously described [22]. The primary and secondary antibodies used in the study are shown in Tables 1 and 2, respectively. Human eye paraffin sections were stained using the protocol previously described [8]. Briefly, antigen retrieval was carried out by boiling slides in antigen retrieval buffer (0.05% citraconic acid, pH 7.4) (Sigma-Aldrich, Cat. C82604) for 30 min and blocked 1 h at room temperature in 10% donkey serum (Sigma-Aldrich, Cat. D9663). Samples were incubated with the primary antibody (Table 1) overnight at 4 °C and 1 h room temperature in the dark with the corresponding secondary antibody (Table 2).

The samples were cover-slipped with DAPI-Vectashield (Vector Labs) and examined by Leica DMi8 epifluorescence microscope or confocal microscope (Leica TCS SP5, Leica Microsystems Ltd., Wetzlar, Germany). The fibrotic lesion was measured in RPE/choroid flatmount using a protocol described previously [22]. The measurements were conducted double-blinded by two independent researchers.

### Collagen matrix contraction assay

Primary RPE cells (1.5 × 10^5^ cells/mL, 500 μL per well) were suspended in 2 mg/mL rat tail type I collagen (Collagen I Rat Protein, Tail A1048301 Thermo Fisher Scientific, Waltham, MA) which was dissolved in 6 μL of 0.1 M NaOH and seeded in a 24-well plate. After incubation at 37 °C for 3 days in DMEM-F12 + 10% FCS, media was changed to DMEM-F12 containing 1% FCS with different treatments and immediately after the cell collagen gels were detached from the bottom of the wells. Pictures of the surface area of each matrix were taken in Syngene G-Box imaging system (Syngene, Cambridge, UK) at 0 h after the detachment and every 24 h thereafter.

### Wound healing assay

Primary RPE cells were seeded in 6-well plates (1.5 × 10^5^ cells per well) and cultured until confluence. The medium was then changed to 1% FCS for 24 h. The cell monolayer was scratched with a 200-µL tip to inflict a wound ∼1 mm in width, washed several times and then treated with C5a in the presence or absence of C5aR antagonist PMX53 in DMEM-F12 with 1% FCS. The wound was photographed immediately and 24 h after the scratch. Images were analysed using ImageJ software (NIH, Bethesda, MD).

### Western blot

Samples were homogenized in RIPA buffer containing protease inhibitor cocktail. Protein concentration was determined using a Pierce BCA protein assay kit (Thermo Fisher Scientific. Cat. 23225) or Bradford Assay (Cat. ab119216, Abcam). The blot was performed using 15 to 20 µg of protein according to previously described methods [26, 27]. Primary and secondary antibodies used are detailed in Tables 1 and 2. Membranes were visualized with enhanced chemiluminescence (Clarity Western ECL Blotting Substrates; Bio-Rad Laboratories) and bands detected using Syngene G-Box imaging system (Syngene). Western Blot analyses were performed using ImageJ software and densitometry normalized to loading control GAPDH or Rab11.

### Enzyme-linked immunosorbent assay (ELISA)

Supernatants of primary murine RPE cells treated with C5a or TGF-β2 for different lengths of time was used for determine the concentration of C5a, C5, TGF-β1, TGF-β2 and VEGF. ELISA kits for C5a, C5, TGF-β1, TGF-β2 and VEGF (R&D Systems), IL-6 and TNF-α (Thermo Fisher Scientific-Invitrogen) (Table 2) were used according to the manufacturers’ instructions. The concentration obtained in pg/mL was then normalized by the total protein concentration of each sample measured with Bradford assay (Cat. ab119216, Abcam).

### Data analysis

We used the Spectacle platform [28] with the dataset from the study of Voigt et al. [29] to analyse the gene expression of epithelial marker E-cadherin (*CDH1*), mesenchymal markers FN (*FN1*) and αSMA (*ACTA2*), C5aR1 (*C5AR1*) and C3aR1 (*C3AR1*) in human RPE/choroidal cells. The dataset in the study by Voigt et al. includes single cell RNA sequencing (ScRNAseq) information of RPE/choroidal tissue from the macular and peripheral areas of healthy donors. The analysis allows identification of specific cell types that express the genes of our interest and the graph is generated by the Spectacle platform.

The Graph Pad Prism (V6, GraphPad Software, San Diego, CA) was used to create graphs and conduct statistical analyses on our laboratory data. Statistical differences between two groups were assessed via an independent Student’s *t* test. On larger datasets, one-way or two-way ANOVA was used where appropriate. Bonferroni correction was used for multiple comparison testing.

## Results

### The effect of complement inhibition in subretinal fibrosis

**Fig. 1:**
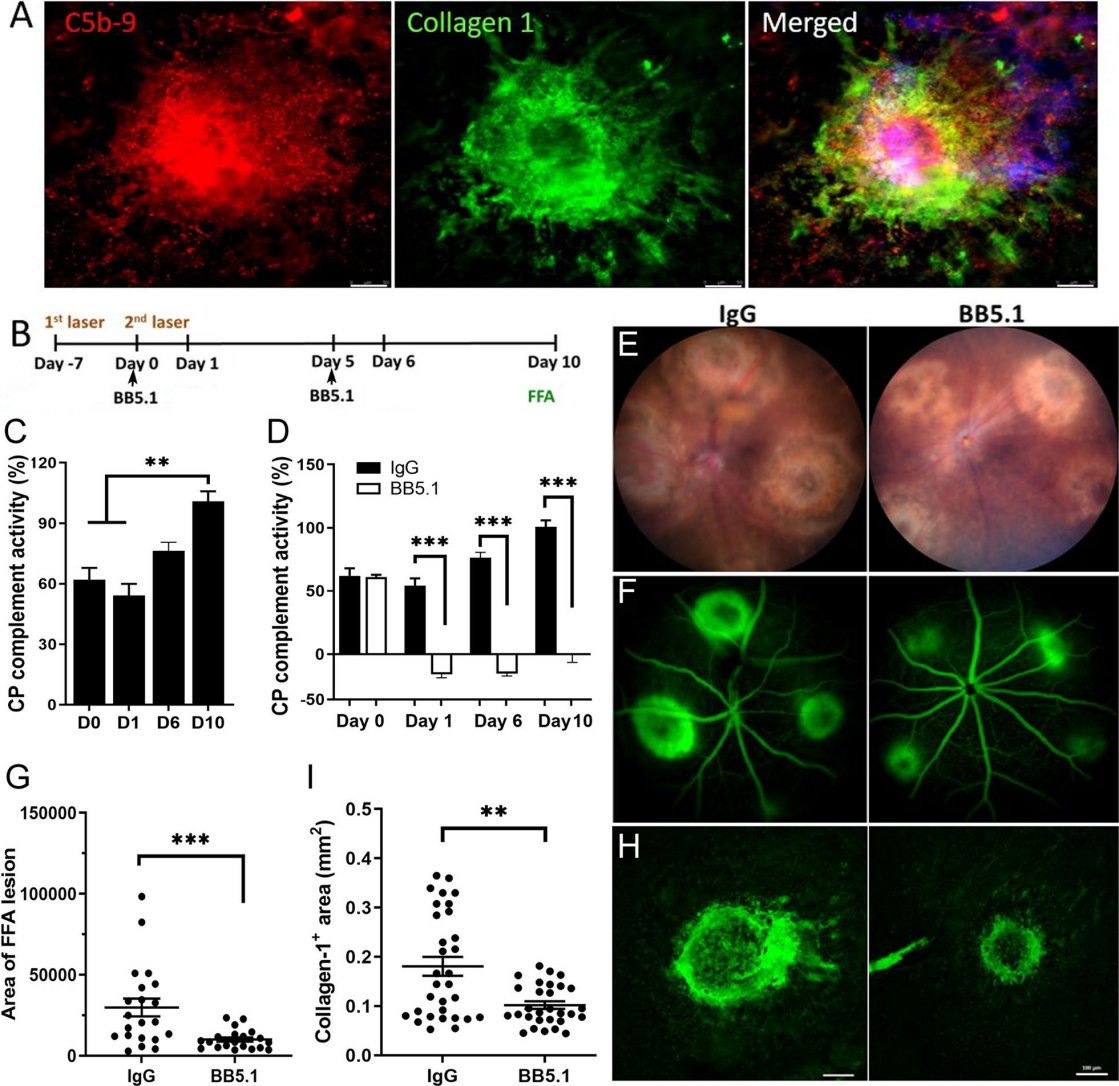
Complement activation in subretinal fibrosis. Subretinal fibrosis was induced in C57BL/6J mice using the two-stage laser-induced protocol (see Methods). **A** Representative epifluorescence images showing C5b-9 deposition in Col-I^+^ subretinal lesion in RPE/choroid flatmount counterstained with DAPI. Scale bar = 100 µm. **B** Schematic diagram showing experimental design of the in vivo C5 blockade study. BB5.1 antibody was administered intraperitoneally on day 0 and 5 after the second laser. Peripheral blood was obtained on days 1, 6 and 10 (end point) for complement activity measurement. Fundus image and fluorescence angiography were carried out immediately before killing the animals on day 10. **C** Complement activity in the serum of subretinal fibrosis mice treated with control IgG. **D** Complement activity in the serum of BB5.1 antibody and IgG-treated mice at different times. Data expressed as % of complement activity. Mean ± SEM, *n* = 3–5 animals. **p < 0.01, one-way ANOVA with Tukey’s multiple comparison tests in **C**; ****p < 0.001 compared to correspondent vehicle controls by multiple *t*-test in **D**. **E**, **F**. Representative fundus colour photography (**E**) and fluorescence angiography (**F**) in IgG and BB5.1-treated mice at day 10 post second laser. **G** Quantitative measurement of the area of fluorescein leakage from fibrosis lesion (in pixels). Mean ± SEM, *n* = 20–21 lesions from 5–6 eyes/group. ***p < 0.005, Student t test. **H** Representative images of Col-I^+^ lesions in RPE/choroid flatmount of IgG (left) and BB5.1 (right) treated mice. Scale bar = 100 μm. **I** Quantitative measurement of Col-I^+^ lesion area in IgG and BB5.1-treated mice. Mean ± SEM, *n* = 29–31 lesions from 8 eyes. **p < 0.01; Mann–Whitney test

To understand if complement activation contributes to the development of subretinal fibrosis, first, we evaluated the expression of C5b-9 complex in the two-stage laser mouse model of subretinal fibrosis. Immunofluorescence detected intense C5b-9 expression in Col-I^+^ fibrotic lesions in RPE/choroidal flatmounts (Fig. 1A). Next, we blocked complement activation using a C5 neutralizing antibody BB5.1 [23] at days 0 and 5 after the second laser (Fig. 1B). A progressive increase in the classical pathway of complement activation in the serum was observed in IgG-treated subretinal fibrosis mice (Fig. 1C). The treatment with BB5.1 completely abolished the classical pathway of complement activation even 10 days after the second laser (Fig. 1D). Clinical examination showed a marked decrease in fluorescein leakage in BB5.1-treated mice compared with IgG-treated controls (Fig. 1E–G). Immunofluorescence of RPE/choroid flatmounts showed that BB5.1 treatment significantly reduced the size of Col-I^+^ fibrotic lesion (Fig. 1H, I). Our results suggest that complement activation significantly contributes to the development of subretinal fibrosis in our model system.

### C5a and C5aR are expressed in retinal pigment epithelial cells

**Fig. 2:**
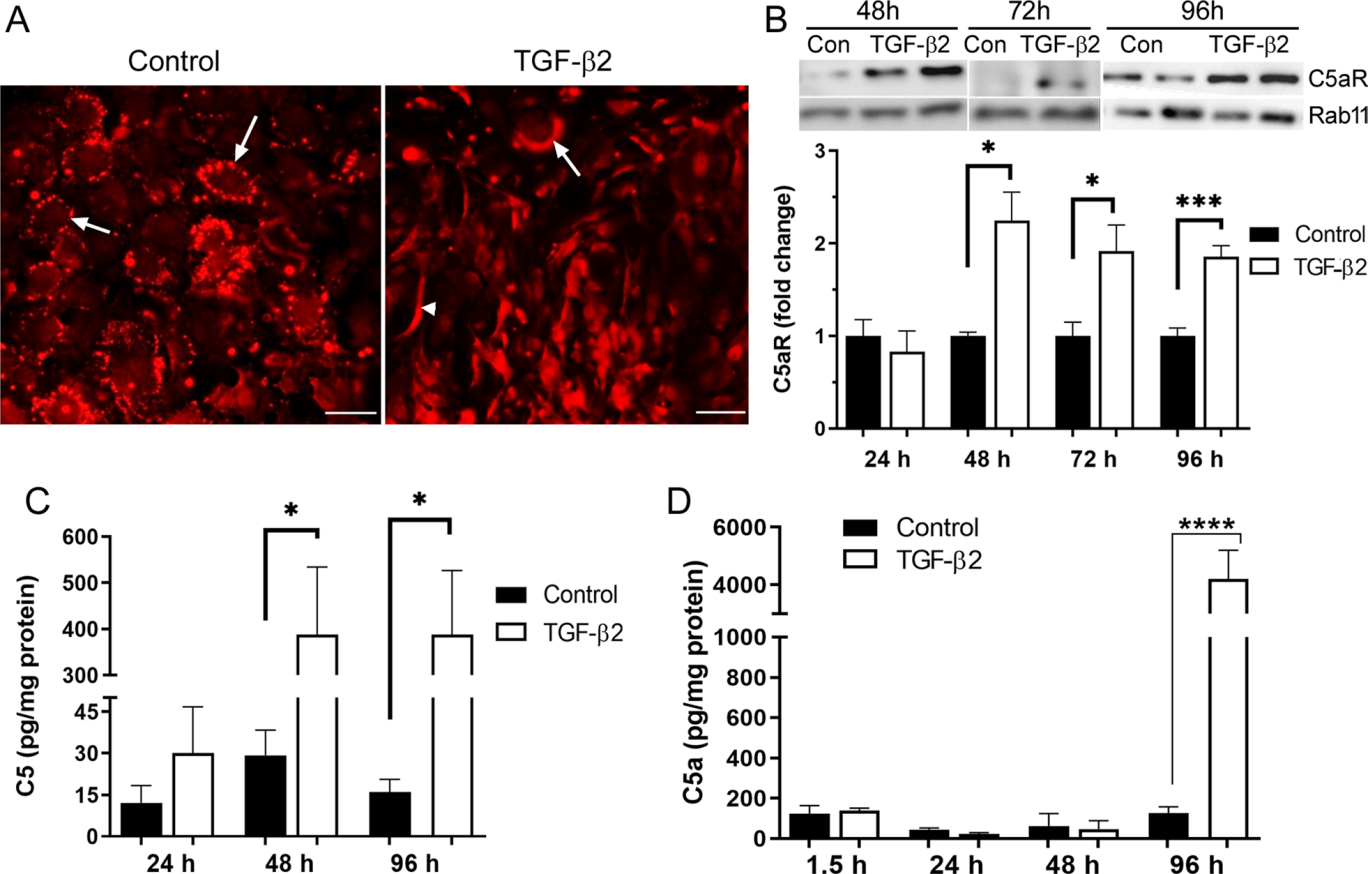
Expression of C5a and C5aR in primary mouse RPE cells. Mouse RPE cells were treated with 10 ng/mL TGF-β2 for different times. **A** C5aR representative immunofluorescence image of RPE cells under normal conditions and after the exposure to TGF-β2 for 48 h. Scale bar = 50 µm. Arrows indicate C5aR expression. Arrowhead, spindle-shaped RPE cells. **B** Western blot analysis of C5aR expression in RPE cells treated with TGF-β2 for different times. Data were representative of 2–3 repeated studies and expressed as fold change of control of each time point. **C**, **D** ELISA quantification of protein concentration of C5 (**C**) or C5a (**D**) in the supernatants of control and TGF-β2-treated RPE cells. Data were normalized to supernatant total protein. Mean ± SEM. *n* **= **3–6. *p < 0.05; ****p < 0.001, Student *t*-test

To understand if the complement system can promote subretinal fibrosis through induction of RPE-to-mesenchymal transdifferentiation, we examined the expression of C5aR and C5a in primary RPE (pRPE) cells with or without TGF-β2 (10 ng/mL) treatment [3]. Immunofluorescence showed punctate C5aR expression in RPE cell membrane under normal culture conditions (arrows, Fig. 2A). Following TGF-β2 treatment, the expression became diffused and extended to cytosols (arrow, Fig. 2A). Multiple spindle shape cells were observed in TGF-β2-treated group (arrowhead, Fig. 2A). Furthermore, Western blot analysis showed a significant increased C5aR expression 48–96 h after TGF-β2 treatment (Fig. 2B). Interestingly, RPE cells also released low levels of C5 (Fig. 2C) and C5a (Fig. 2D) under normal culture conditions. TGF-β2 treatment significant increased C5 production after 48 h (Fig. 2C) and C5a production after 96 h (Fig. 2D).

### C5a, but not C3a, induced epithelial-to-mesenchymal transition in retinal pigment epithelial cells

**Fig. 3:**
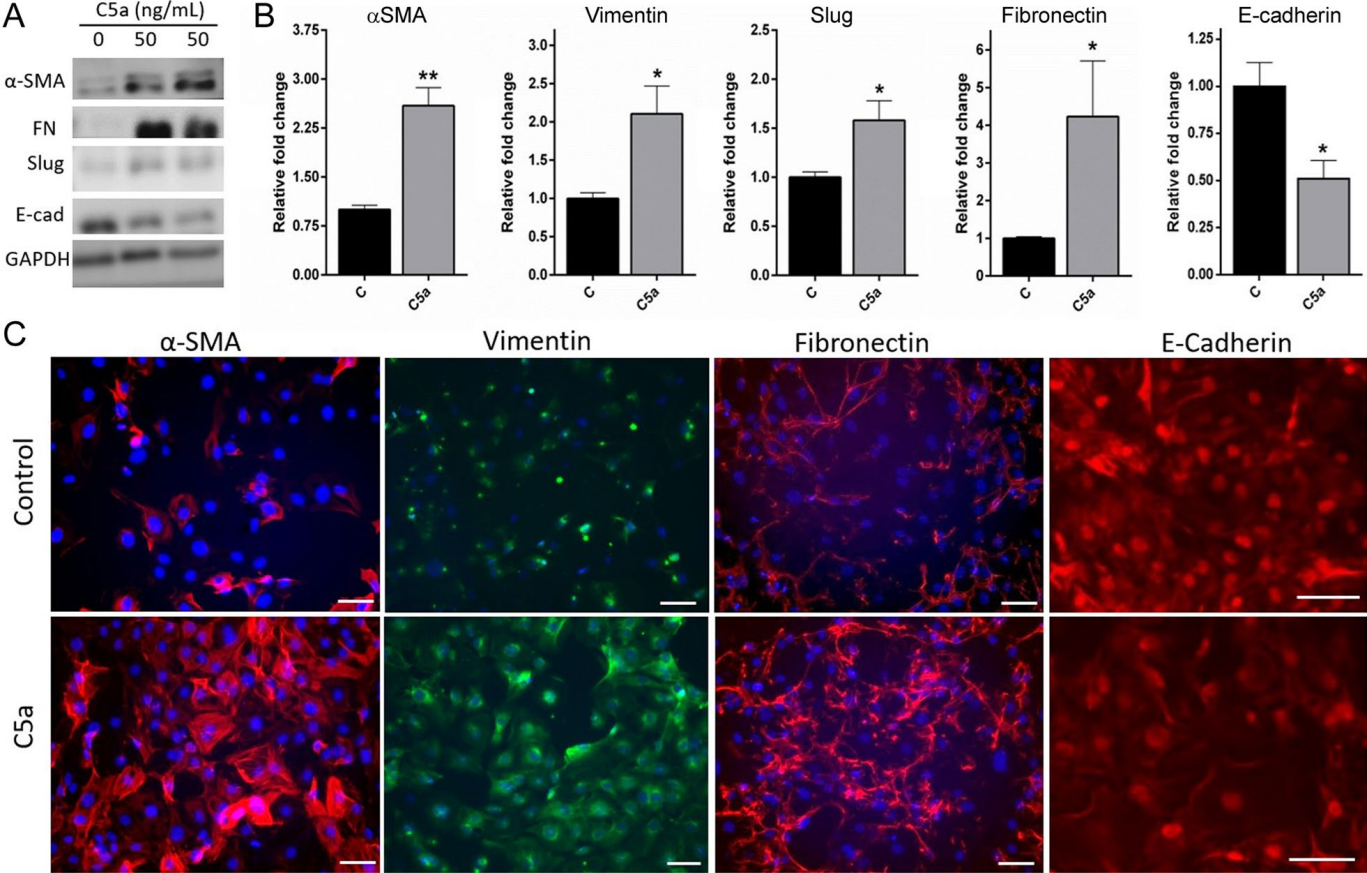
Effect of C5a on RPE cell epithelial-to-mesenchymal transition. Primary mouse RPE cells were treated for 96 h with 50 ng/mL of recombinant C5a. Cells were collected and processed for investigations of mesenchymal and epithelial markers by Western blot (**A**, **B**) and immunocytochemistry (**C**). **A** Representative blot of α-SMA, vimentin, Slug or Fibronectin (FN), and E-cadherin (E-cad) in control and C5a-treated RPE cells. **B** Quantitative data of Western blot from 2 to 3 independent experiments (1 ~ 2 batches of RPE cells/experiment). Means ± SEM. *p < 0.05; **p < 0.01, Student *t*-test. *n* = 3–6. **C** Immunocytochemistry of α-SMA, Vimentin, FN and E-cad in control and C5a-treated RPE cells. Scale bar = 50 μm

Having shown that RPE cells constitutively express C5aR and release C5/C5a, we then investigated whether activation of the C5aR could lead to RPE-to-mesenchymal transdifferentiation. Mouse primary RPE cells were treated with different concentrations of C3a and C5a [8, 16]. A time-course study showed that treatment of RPE cells with C5a (50 ng/mL) for 24–72 h did not elicit significant changes in the expression of the mesenchymal marker FN (Additional file 1: Fig. S1A, B) or the epithelial marker E-Cadherin (Additional file 1: Fig. S1A, C). However, 96-h treatment with C5a significantly increased mesenchymal marker FN and decreased epithelial marker E-cadherin expression (Additional file 1: Fig. S1A–C). Among different concentrations, 50 ng/mL was the lowest concentration that elicited significant upregulation of FN and downregulation of E-cadherin (Additional file 1: Fig. S1). C5a-induced EMT was also evidenced by the morphological alteration of RPE cells from cobblestone-like morphology to elongated spindle shape (Additional file 1: Fig. S1G). Therefore, we used 50 ng/mL of C5a in subsequent studies. Western blot showed other markers of EMT including αSMA, vimentin and the transcription factor Slug were significantly upregulated alongside with FN after 96 h of C5a treatment (Fig. 3A, B). The results were further confirmed by immunofluorescence staining (Fig. 3C).

C3a treatment (10–100 ng/mL, up to 96 h) did not significantly alter the expression levels of FN and α-SMA in RPE cells (Additional file 1: Fig. S2A, B). We previously showed that 10 ng/mL of C3a induced macrophage-to-myofibroblast transition [22]. Using this concentration of C3a for 96 h, neither E-cad expression (Additional file 1: Fig. S2C) nor the collagen contractility were affected in RPE cells (Additional file 1: Fig. S2D). Interestingly, when C3a was used in combination with C5a, it blocked C5a-mediated downregulation of E-cadherin and upregulation of α-SMA and FN in RPE cells (Additional file 1: Fig. S3). A previous study has shown that the engagement of C3aR on RPE cells could prevent further C5aR responses [30], which is in line with our observation. Nevertheless, our results suggest that C5a, but not C3a can drive EMT in RPE cells.

### C5a-induced EMT is mediated by C5aR

**Fig. 4:**
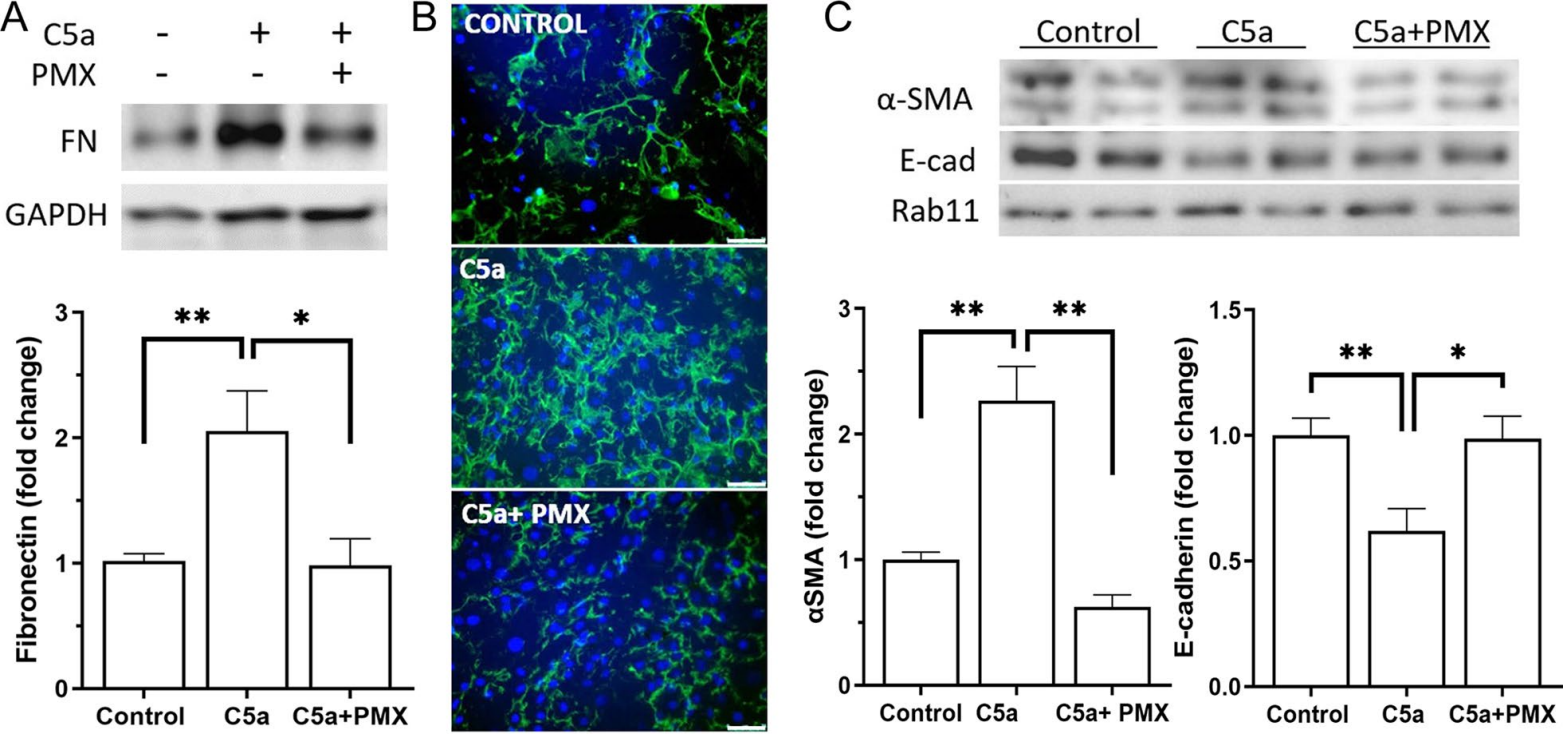
The role of C5aR in C5a-induced EMT in RPE cells. Primary mouse RPE cells were treated for 96 h with recombinant C5a (50 ng/mL) with or without C5aR antagonist PMX53 (50 nM). Changes in fibronectin (FN) expression were determined by Western blot (**A**) and confirmed by immunofluorescence (**B**). **C**, Representative Western blot images and densitometry analysis of αSMA and E-cadherin (E-cad) protein expression in control, C5a and C5a + PMX53-treated RPE cells. Means ± SEM from 2 independent experiments (2 ~ 3 batches of RPE/experiment). *n* = 4–6. *p < 0.05; **p < 0.01, one-way ANOVA followed by Tukey’s multiple comparison tests

To understand if C5a-induced EMT in RPE cells is mediated through its receptor C5aR, PMX53, a C5aR-specific antagonist was used [31–33]. Co-treatment with PMX53 (50 nM) prevented C5a-induced upregulation of FN, as shown by Western blot (Fig. 4A) and immunocytochemistry (Fig. 4B). In addition, C5a-induced upregulation of α-SMA and downregulation of E-cadherin in RPE cells were also prevented by PMX53 treatment (Fig. 4C).

**Fig. 5:**
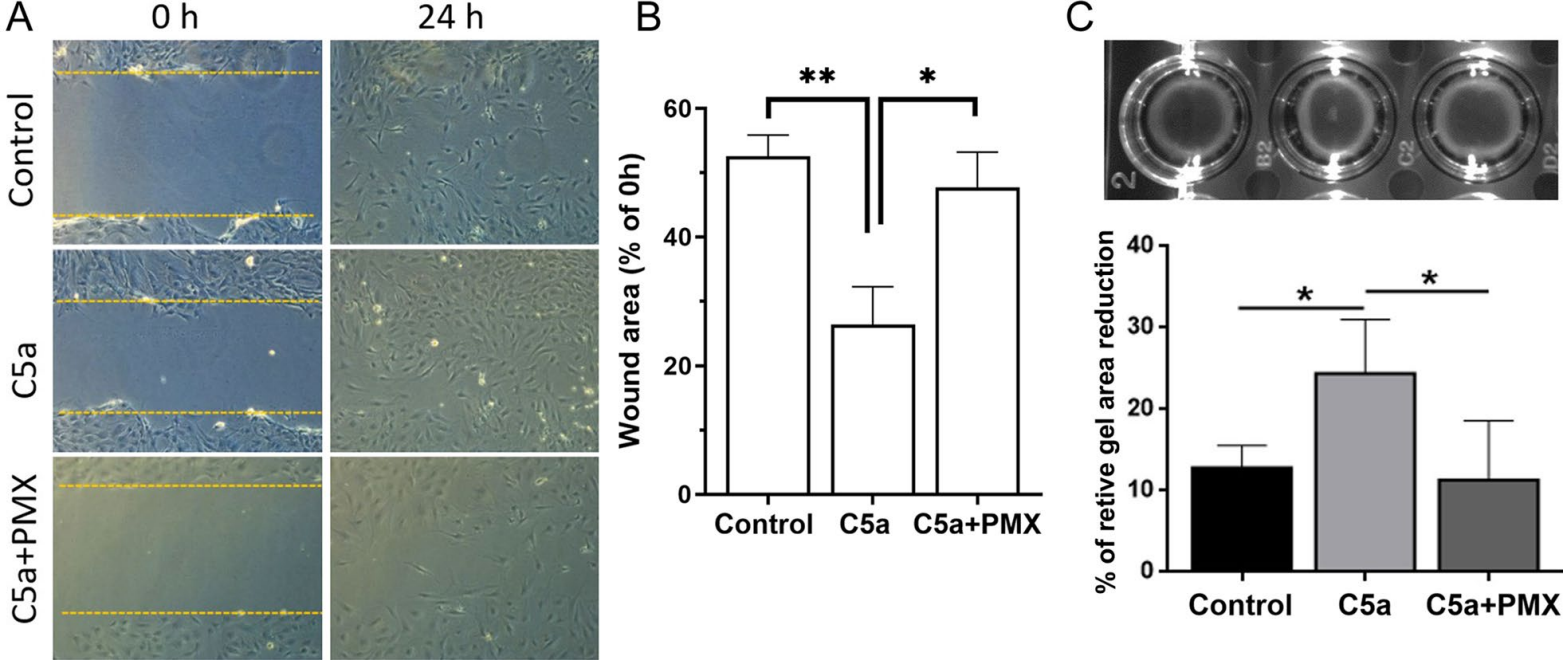
The effect of C5a on RPE wound-healing and contractility. Primary mouse RPE monolayers were subjected to a 1 mm width scratch. Cells were then exposed to C5a (50 ng/mL) for 24 h with or without PMX53 (50 nM). **A** Representative images from control, C5a or C5a + PMX53-treated groups immediately (0 h) and 24 h after wound scratch. **B** Changes in the wound area covered by the cell monolayer in different groups. Data expressed as % of remaining wound area. **C** Mouse RPE cells cultured in collagen gels were treated with recombinant C5a in the presence or absence of PMX53 for 24 h. The gel contraction was measured at the end of the experiment. Data expressed as % of reduction in gel area. *n* = 4. *p < 0.05; **p < 0.01; one-way ANOVA followed by Tukey’s multiple comparison tests. The wound scratch assay was conducted twice and the collagen gel contraction assay was conducted four times and each with different batches of pRPE cells

We used a wound healing assay to determine the invasiveness of RPE cells undergoing EMT. C5a treatment (50 ng/mL) significantly promoted RPE wound healing (Fig. 5A, B) and this effect was prevented by PMX53 (Fig. 5A, B). In the collagen gel contraction assay, C5a pre-treated RPE cells significantly increased gel contractility, and this was inhibited by PMX53 (Fig. 5C). C3a pre-treated RPE cells did not affect collagen gel contractility (Additional file 1: Fig. S2D).

### C5a induced the release of pro-inflammatory and pro-fibrotic mediators

**Fig. 6:**
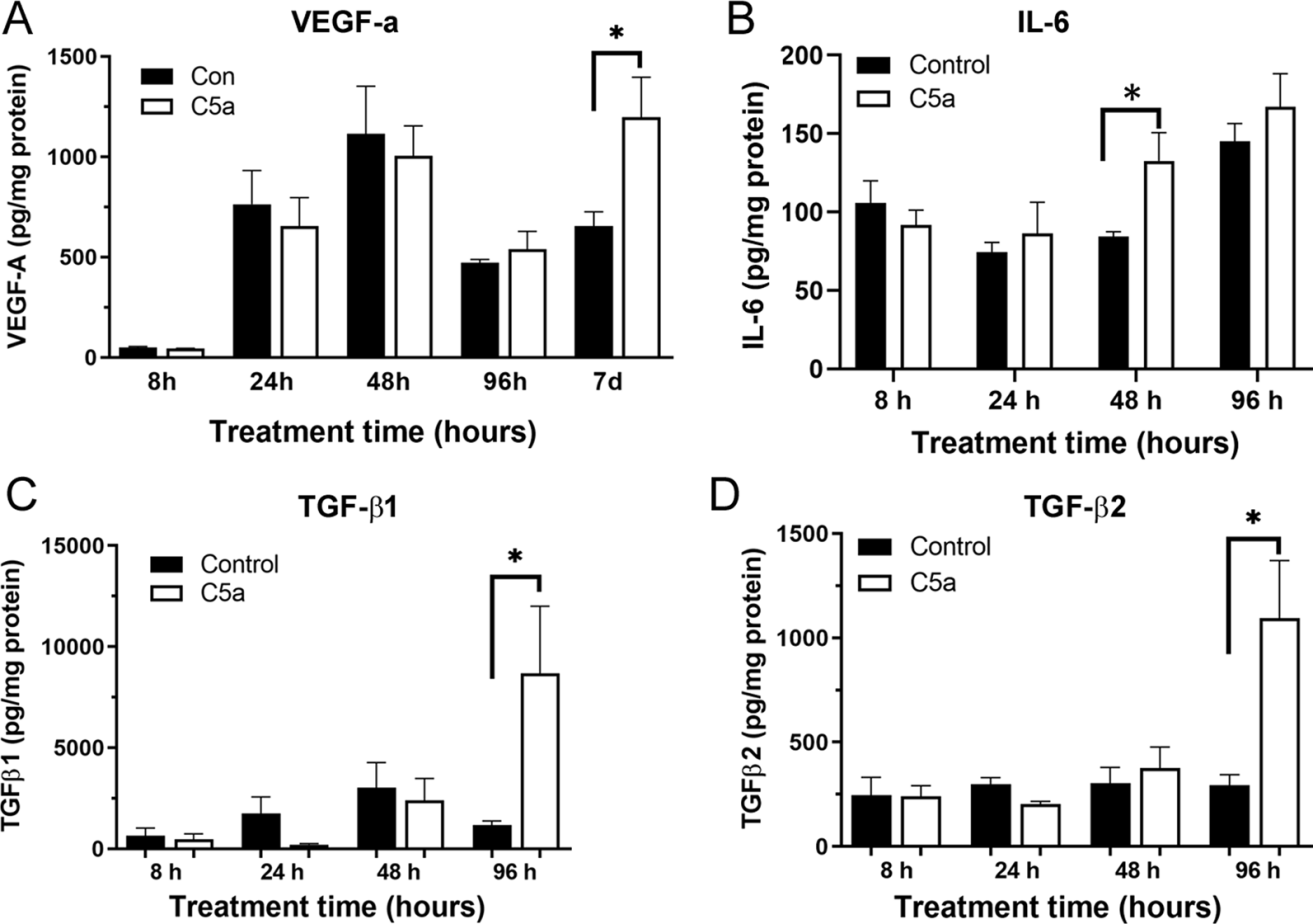
The effect of C5a on RPE cell pro-fibrotic mediator production. Primary mouse RPE cells were treated with C5a (50 ng/mL) for different times. The supernatants were collected for ELISA analysis of VEGF-a, IL-6, TGF-β1, and TGF-ß2. **A** VEGF-A production in control and C5a-treated RPE cells at different times. (B) IL-6 production in control and C5a-treated RPE cells at different times. **C**, **D** The production of TGF-β1 (**C**) and TGF- β2 (**D**) in control and C5a-treated RPE cells at different times. Mean ± SEM, N = 4–6, *p < 0.05, Student *t*-test

To further understand the effect of C5a on RPE cells, we examined the production of VEGF-A, IL-6, TGF-β1 and TGF-β2, and TNF-α by pRPE cells following C5a treatment. As shown in Fig. 6, VEGF-A production was not affected by C5a until 7 days after the treatment. The level of IL-6 was slightly higher in 48 h C5a-treated cells compared control cells from the same time point (Fig. 6B). The production of TGF-β1 and TGF-β2 was significantly increased after 96 h treatment with C5a (Fig. 6C, D). TNF-α in pRPE supernatant was undetectable by ELISA at any point with/without C5a treatment (data not shown). During chronic inflammation, IL-6 can drive fibrosis [34]. VEGF-A is known to induce pro-fibrotic growth factor and extracellular matrix gene expression in the retina in vivo and in cultured retinal vascular endothelial cells [35]; whereas, TGF-β is the master regulator of fibrosis [36]. In the presence of IL-6, TGF-β can induce Th17 cell differentiation. Therefore, our results suggest that C5a drives RPE cells into a pro-inflammatory and pro-fibrotic state.

### The effect of C5a on pSmad2/3 and ERK expression in RPE cells

**Fig. 7:**
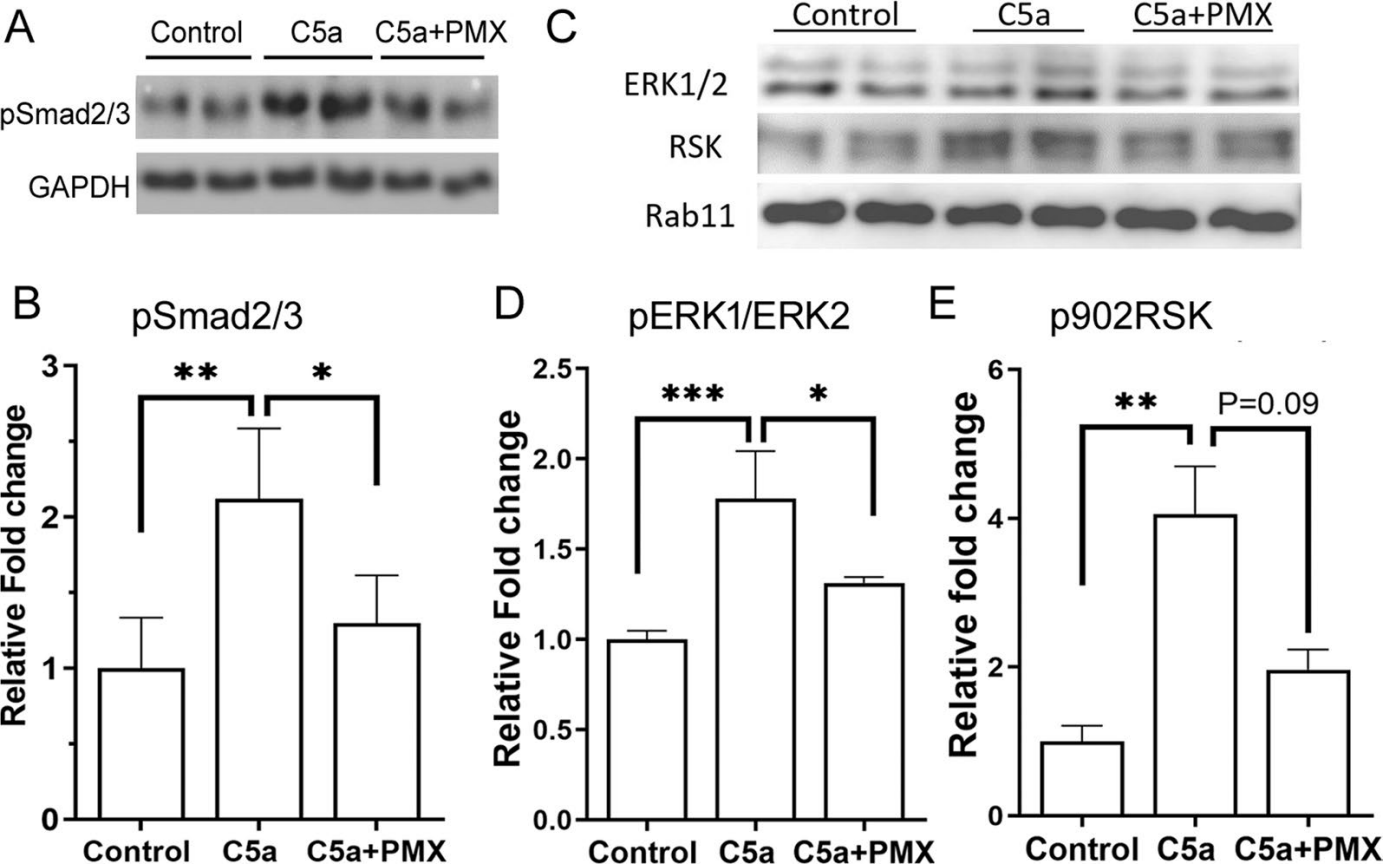
The effect of C5a on Smad2/3 and ERK expression in RPE cells. Primary mouse RPE cells were treated with C5a (50 ng/mL) for 24 h with or without PMX53 (50 nM). The expression of pSmad2/3, ERK, and p90RSK was determined by Western blot. **A** Representative Western blot images of pSmad2/3. **B** Quantification of pSmad2/3 expression in different groups. **C** Representative Western blot images of ERK1/2 and p90RSK. The pERK antibody recognizes pERK1 (Y204) + pERK2 (Y197). **D** Quantification of pERK1/2 expression in different groups. **E** Quantification of p90RSK (S380) expression in different groups. Mean ± SEM, *n* = 4–6 (from 2 ~ 3 independent studies). *p < 0.05; **p < 0.01; ***p < 0.005, ANOVA one-way followed by Tukey’s post hoc test

We next examined the signalling pathways responsible for C5a-induced RPE changes, including the pSmad2/3, pERK1/2 and RSK that are known to be involved in the EMT processes [3, 7]. Our results show that C5a treatment (50 ng/mL, 24 h) significantly upregulated the expression of phosphorylated Smad2/3 in RPE cells and this effect was reduced by PMX53 (Fig. 7A, B). C5a treatment also increased the phosphorylation of ERK1/2 in the tyrosine 204/197 and the p90 ribosomal protein S6 kinase (p90RSK) in Serine 380 (Fig. 7C). PMX53 treatment significantly attenuated C5a-induced pERK1/2 but not p90RSK expression (Fig. 7C–E).

### C5aR expression in mouse subretinal lesions

**Fig. 8:**
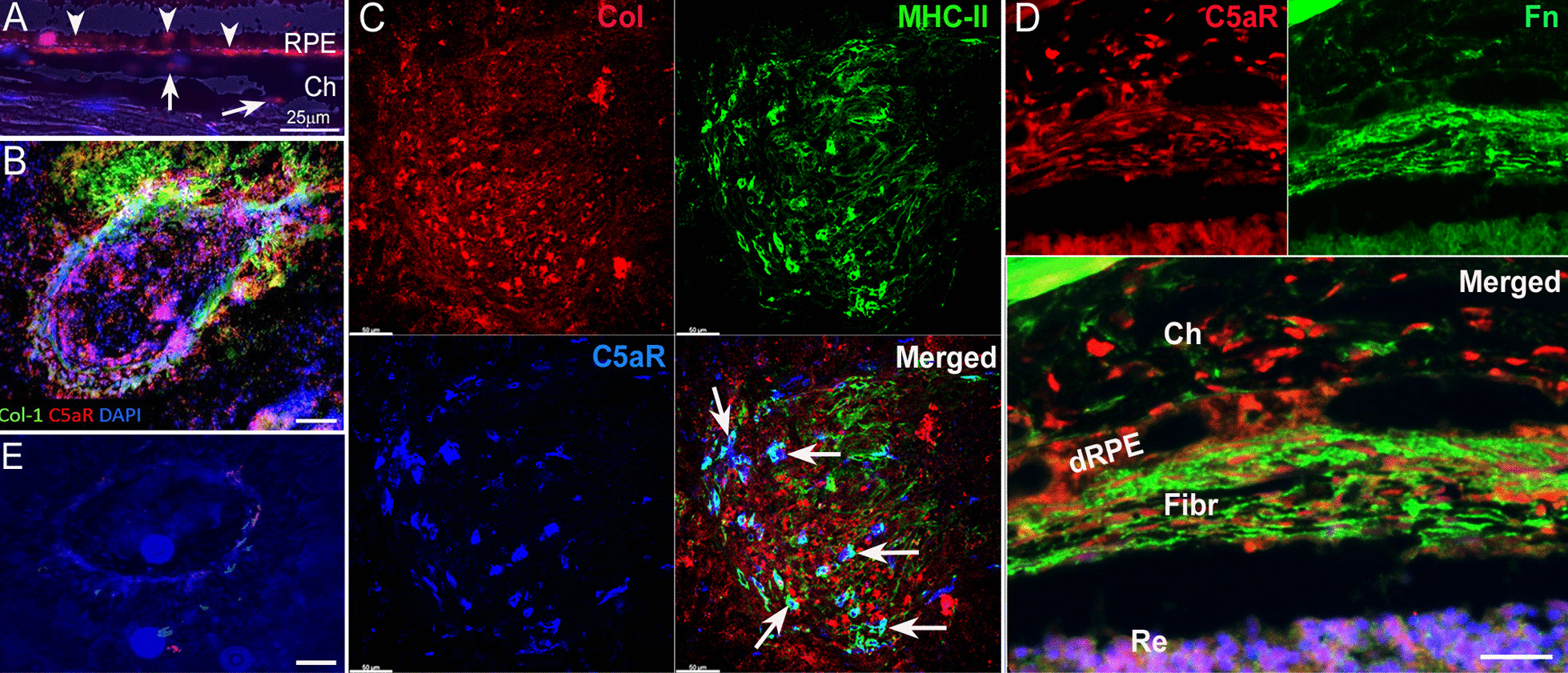
C5aR expression in the subretinal fibrotic lesion in mouse eyes. **A** Representative image of normal mouse eye section showing C5aR (red) expression in RPE cells (arrowheads) and choroidal cells (arrows). **B** Representative image of a RPE/choroid flatmount from day 10 post the second laser mouse eye stained for Collagen-I (Col-1, green), C5aR (red) and DAPI. **C** A confocal image showing Col-1 (red), MHC-II (green) and C5aR (blue). Arrows indicate MHC-II^+^C5aR^+^ cells. **D** A confocal image of mouse eye section 20 days after the second laser stained for fibronectin (Fn, green), C5aR (red) and DAPI showing C5aR (red) expression in subretinal fibrotic lesion. **E** Isotype control staining of a RPE/choroidal flatmount showing no immunoreactivity of collagen-1 and C5aR. Scale bar = 50 µm. Col: collagen-1; Fn: fibronectin; Ch: choroid; dRPE: damaged retinal pigment epithelial layer; Fibr: fibrotic lesion; Re: retina

To understand if the in vitro effects of C5a on RPE cells is relevant to subretinal fibrosis in vivo, we investigated the expression of C5aR in fibrotic tissues. In normal mouse eye, C5aR was detected in RPE, particularly at the basal side/Bruch’s membrane as well as in choroidal cells (Fig. 8A). In the two-stage laser-induced mouse model of subretinal fibrosis, C5aR was detected in Col-I^+^ lesions in RPE/choroid flatmounts (Fig. 8B). Co-staining of MHC-II and C5aR indicated that the majority of infiltrating MHC-II^+^ immune cells expressed C5aR (arrows in Fig. 8C, merged image). Investigation of ocular tissue sections revealed extensive C5aR expression in the choroid, damaged RPE cells and inside the fibrotic lesions (Fig. 8D). Isotype control staining did not show any immunoreactivity of collagen-2 and C5aR in subretinal fibrotic lesion (Fig. 8E).

### The effect of C5aR blockade on subretinal fibrosis

**Fig. 9:**
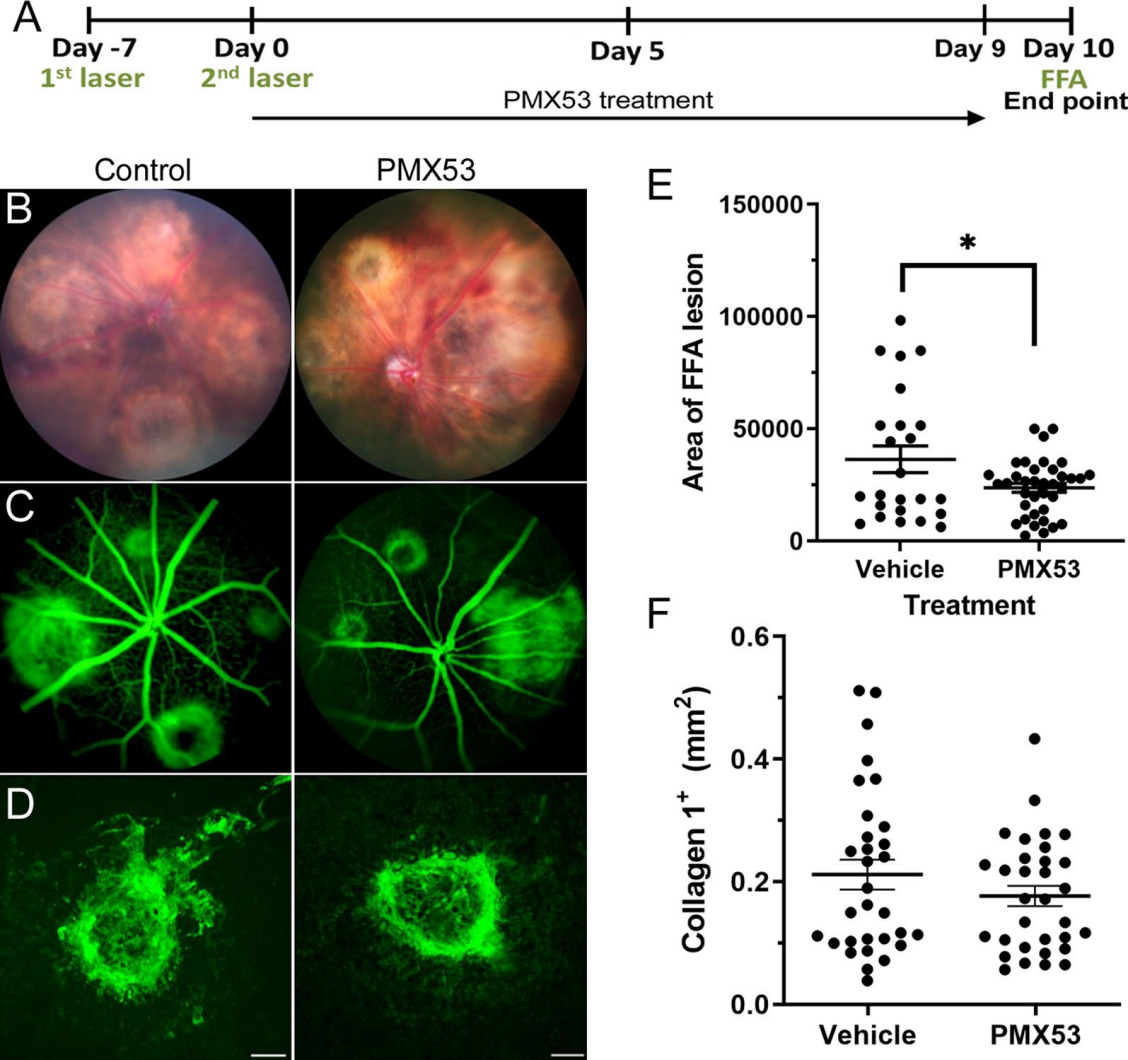
Effect of C5aR blockade on a mouse model of subretinal fibrosis. Subretinal fibrosis was induced in C57BL/6J mice using the two-stage laser-induced protocol (see Methods). **A** Schematic diagram shows experimental design of the in vivo C5aR blockade study. PMX53 (1 mg/kg) was administered subcutaneously daily from day 0 until day 9. On day 10, mice were subjected to fundus imaging and fluorescence angiography before killing the animals. **B**, **C** Representative fundus colour photography (**B**) and fluorescence angiography (**C**) in PMX53 and vehicle-treated (control) mice at day 10 post second laser. (**E**) Quantitative measurement of the area of fluorescein leakage from fibrosis lesion (in pixels). Mean ± SEM, *n* = 24–30 lesions from 8–9 eyes/group. *p < 0.005, Mann–Whitney test. **D** Representative images of Col-I^+^ lesions in RPE/choroid flatmount of vehicle and PMX53-treated mice. Scale bar = 100 μm. **F** Quantitative measurement of collagen-I^+^ lesion area in vehicle and PMX53-treated mice. Mean ± SEM, *n* = 30 lesions from 8 eyes

To evaluate the role of the C5a/C5aR pathway in the development of subretinal fibrosis, we blocked C5aR with PMX53 in the two-stage laser model of subretinal fibrosis. Animals were treated with PMX53 daily (s.c.) immediately after the 2nd laser for 9 days and killed at day 10 (Fig. 9A). Fundus fluorescence angiography showed a significantly decreased area of leakage in PMX53-treated mice compared to the vehicle controls (Fig. 9B, C, E). Col-I^+^ fibrotic lesions in the RPE/choroid flatmounts were marginally smaller in PMX53-treated mice compared to vehicle-treated mice (0.21 ± 0.02 vs. 0.17 ± 0.016 mm^2^, *p* = 0.11) (Fig. 9D, F). Our results suggest that C5a–C5aR may partially contribute to the development of subretinal fibrosis.

### C5aR pathway in macular fibrosis of human nAMD

**Fig. 10:**
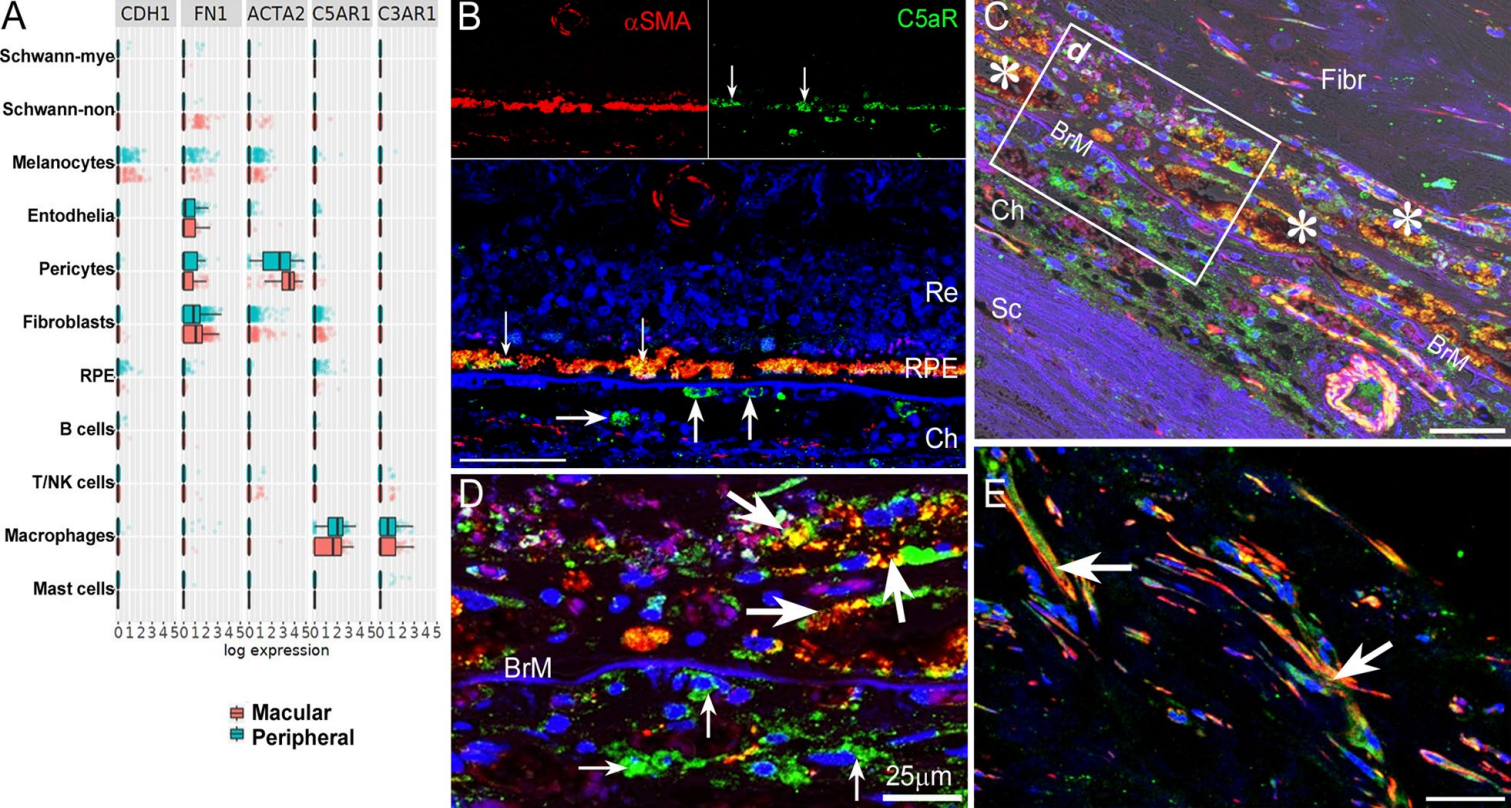
Epithelial/Mesenchymal marker expression in human eyes. **A** The expression of *CDH1, FN1, ACTA2, C5AR1* and *C3AR1* in different types of choroidal cells from macular and peripheral area identified by the Spectacle platform [28] using the dataset of dataset of Voigt et al. [29]. **B**–**E** Immunohistochemistry of human paraffin sections from the non-lesion area (B) and macular fibrosis lesion area (**C**–**E**) of a nAMD donor eye stained for C5aR (green) and α-SMA (red) and DAPI. Thin arrows in **B** indicate C5aR expression in RPE cells. Small arrows in B and D indicate choroidal C5aR^+^αSMA^−^ cells. Asterisks in C indicating clumps of pigmented cells that were C5aR^+^α-SMA^+^. **D** High-magnification view of the rectangle area in **C**. **E** C5aR and α-SMA expression inside the fibrotic lesion. Big arrows in **D** and **E** indicate C5aR^+^ α-SMA^+^ cells. Scale bar (**D**, **E**) = 50 µm. BrM: Bruch’s membrane; Fibr: fibrosis; Ch: choroid; RPE: RPE; Re: retina; Sc: sclera

To understand if our study is relevant to macular fibrosis in nAMD patients, we investigated the gene expression of epithelial marker E-cadherin (*CDH1*), mesenchymal markers FN (*FN1*) and α-SMA (*ACTA2*), C5aR1 (*C5AR1*) and C3aR1 (*C3AR1*) in RPE/choroidal cells using the Spectacle [28] with the dataset of Voigt et al. [29]. *CDH1* was expressed predominately by melanocytes and RPE cells (Fig. 10A). *FN1* and *ACTA2* were expressed by melanocytes, pericytes, fibroblasts, endothelial cells, non-myelin producing Schwann cells and some RPE cells (Fig. 10A). *C5AR1* was detected in macrophages, fibroblasts and RPE cells (Fig. 10A). The expression levels of *C5AR1* in peripheral RPE appear to be higher than macular RPE (Fig. 10A). *C3AR1* was detected only in choroidal macrophages and a small population of T/NK cells (Fig. 10A). Immunofluorescence of human ocular sections showed that C5aR was positive in choroidal cells (likely macrophages, arrows in Fig. 10B) and RPE cells (small arrows, Fig. 10B) in non-fibrosis area. In the area with macular fibrosis, RPE cells were detached from Bruch’s membrane (BrM) and formed clumps either on top of BrM or inside the fibrotic lesion (asterisks, Fig. 10C), and the pigmented cells were positive for α-SMA (red) and C5aR (green) (Fig. 10C, D, big arrows). Many choroidal C5aR^+^ cells were negative for α-SMA in the choroid (small arrows in Fig. 10D). A large number of elongated αSMA^+^C5aR^+^ cells were detected inside the fibrotic lesion (big arrows in Fig. 10E), likely transdifferentiated myofibroblasts. Our results suggest the existence of the C5aR pathway in macular fibrosis secondary to nAMD.

## Discussion

The development of subretinal fibrosis following CNV is associated with multiple biological processes including infiltration of inflammatory cells such as macrophages [8], EMT of RPE cells, EndoMT and recruitment of fibrocytes from blood and choroid [7]. The molecular cues that are involved in the conversion of CNV into a fibro-vascular lesion remain poorly defined. In this study, we show that complement activation critically contributed to the development of subretinal fibrosis and blocking complement activation with BB5.1 significantly reduced subretinal fibrosis. Mechanistically, the complement system may promote subretinal fibrosis, at least partially, through C5a-induced EMT in RPE cells.

Previously, we reported higher plasma levels of C3a and C5a in patients with macular fibrosis. The circulating complement fragments (C3a and C5a) may be recruited to the diseased macula and participate in fibrosis development. C3a and C5a can also be released locally from cleavage of C3 and C5 during complement activation in AMD. We found that RPE cells constitutively produced C5/C5a and the production was enhanced by TGF-β2 (Fig. 2C, D), suggesting that RPE-derived C5 may contribute to complement activation in retinal fibrosis. C5a is a potent pro-inflammatory peptide and can act as an anaphylatoxin as well as a chemoattractant for various immune cells including neutrophils, eosinophils, monocytes, and T lymphocytes [37]. C5a interacts with its receptor C5aR (CD88), a G-protein-coupled receptor capable of modulating cell function and behaviour. The C5a/C5aR pathway is known to be involved in the pathogenesis of various retinal diseases, including uveoretinitis [23] and AMD [38, 39] through (a) C5a-mediated recruitment of circulating immune cells [39] and (b) C5a-induced inflammatory responses in macrophages [23] and RPE cells [40]. Here, we show that C5aR, but not C3aR is expressed in RPE cells and activation of the C5a/C5aR pathway led to EMT in RPE cells, a phenomenon that has not been reported before. C5a dose-dependently upregulated the expression of myofibroblast marker FN and down-regulated epithelial marker E-cadherin in RPE cells. The mesenchymal phenotype of C5a-treated RPE cells was further confirmed by their expression of α-SMA, vimentin, the transcription factor Slug (Fig. 3) and their high migration and contraction activities (Fig. 5). It is well-known that TGF-β can induce EMT in RPE cells. Interestingly, we found that the production of C5/C5a in RPE cells was enhanced by TGF-β, and the treatment of RPE with C5a also stimulated the release of TGF-β1 and TGF-β2, suggesting a positive feedback loop between C5a- and TGF-β-induced EMT in RPE cells.

The complement system is known to be involved in tissue/organ fibrosis including the lung [13, 14], kidney [15–18], liver [20] and retina [8]. C3a and C5a can induce mesenchymal transition in macrophages [8], pericytes [17], epithelial cells [15, 16, 41] and endothelial cells [18]. Complement activation is critically involved in the development of choroidal neovascularization (CNV) [12, 42]. Parsons et al. suggested that persistent complement activation may be required to maintain the fibrotic scar in CNV, since blocking the alternative pathway of complement activation accelerates retinal repair beyond the normal rate [42]. Here, we show for the first time, that blocking complement activation using a C5 neutralizing antibody significantly reduced subretinal fibrosis secondary to CNV. Apart from RPE cells, C5aR is also expressed in choroidal macrophages and fibroblasts (Figs. 8 and 10). It has been reported that C5a can stimulate macrophage polarization towards alternatively activated pro-fibrotic M2 phenotype [43]. C3aR, on the other hand, is expressed predominately in choroidal macrophages (Fig. 10A). We previous showed that C3a but not C5a induced macrophages-to-myofibroblast transition (MMT) [8]. It is possible that C3a-induced MMT, and C5a-induced M2 macrophage polarization and EMT in RPE cells shown here, may all contribute to complement-mediated subretinal fibrosis. In line with previous reports [17, 18], we found that C5a-induced EMT in RPE involves the canonical TGF-β pathway (Smad2/3) as well as the non-canonical, the GCPR receptor C5aR pathway (ERK1/2).

A sustained low-grade inflammation in the vascular lesion site is known to be a crucial driver of macular fibrosis [44]. In addition to MMT and EMT, the complement proteins may also promote macular fibrosis through the induction of pro-inflammatory and pro-fibrotic factors in macrophages and RPE cells. A previous study reported increased expression of IL-8, IL-1β, IL-6, GM-CSF, and CCL2 (MCP-1) in C5a-treated RPE cells [40]. Here, we found the production of TGF-β1/2 and IL-6 was increased in C5a-treated RPE cells, in line with previous observations in renal tubular epithelial cells and macrophages [16, 45]. C5a may drive RPE cells into a pro-inflammatory state in the process of transdifferentiating from epithelial to mesenchymal phenotype. These pro-inflammatory and pro-fibrotic mediators can initiate a cascade of cellular changes and induce myofibroblast transition from other cells such as macrophages [8], Müller cells [46] or endothelial cells [7] to further contribute to subretinal fibrosis.

Deletion or blockade of C5aR has been found effective in alleviating tubulointerstitial [15, 45], pancreatic [47], glomerular [18] and pulmonary [13, 48] fibrosis. In our study, the administration of the C5aR antagonist PMX53 significantly reduced subretinal fibrosis in clinical examination, but less so in immunohistochemical examination. Instead, blocking the overall complement activation (with BB5.1) strongly suppressed subretinal fibrosis. The lack of efficacy of PMX53 in in vivo fibrosis (but not in in vitro EMT) has been observed in arthritis [49, 50], and possible explanations may include: (1) PMX53/Mas-related gene 2 (MrgX2) mediated mast cell degranulation [32]. Mast cell degranulation and/or the associated histamine release has been shown to be involved in the pathogenesis of various organ fibrosis [51, 52]; (2) rapid elimination of the drug or its poor tissue penetrance [31, 33, 50]; (3) C5a–C5aR2 (C5L2) mediated inflammatory response. C5aR2 is another C5a receptor that is thought to regulate the C5a–C5aR effects although C5aR2 signalling function (anti- or pro-inflammatory properties) is contradictory [53].

## Conclusions

This study suggests that complement activation critically contributes to subretinal fibrosis secondary to nAMD through C5a/C5aR-mediated EMT in RPE cells, which is likely to work in parallel with C3a-induced MMT in macrophages [8]. C5a may also promote macular fibrosis by activating choroidal macrophages and fibroblasts. Blocking complement activation in combination with existing VEGF inhibition could be a novel approach to prevent or reduce macular fibrosis in nAMD.

## Supplementary Information

**Additional file 1: Figure S1**. The effect of C5a on the expression of fibronectin (FN) and E-cadherin (E-cad) in RPE cells. Primary mouse RPE cells were treated with C5a (50 ng/mL) for different times (A–C) or with different concentrations for 96 h (D-F) and collected for Western blot. (A) Representative Western blot images. (B, C) Quantification of FN (B) and E-cad (D) expression on murine RPE treated with or without C5a for different times. (D) Representative Western blot images from RPE cells treated with different concentrations of C5a (10–100 ng/mL) for 96 h. (E, F) Quantification of FN (E) and E-cad (F) expression on RPE treated with different concentrations of C5a for 96 h. Means ± SEM, *n* = 3–6 from 2 independent experiments. *p < 0.05 compared to control (0 h); one-way ANOVA followed by Dunnett post hoc test. (G) Representative phase-contrast images of pRPE cells from control, C5a (96 h) and TGF-β2 (96 h) treated group. Scale bare = 50 µm. **Figure S2.** Effect of C3a on RPE cell epithelial-to-mesenchymal transition. (A, B) Primary murine RPE cells were treated with different concentrations (10, 50, 100 ng/mL) of C3a for 96 h. (C) E-cadherin expression was determined after 96 h treatment with 10 ng/mL of C3a by Western Blot. Mean ± SEM, *n* = 3–6. (D) Changes in contractility were evaluated in RPE cells after 48 h of C3a (10 and 50 ng/mL) treatment. TGF-β2 (10 ng/mL) was used as a positive control. The area of collagen gel in each group was measured and results were expressed as % of reduction in gel area. Means ± SEM, n** = **3–5 gels from 2 independent experiments. ****p < 0.001; one-way ANOVA followed by Dunnett post hoc test compared with untreated. **Figure S3.** The effect of concomitant treatment of primary mouse RPE (pRPE) cells with C5a and C3a. Murine pRPE cells were treated for 96 h with C3a (10 ng/mL) and C5a (50 n/mL). The expression of mesenchymal markers α-SMA and FN, along with epithelial marker E-cadherin was examined by Western Blot. *n* = 2–4. *p < 0.05; **p < 0.01, one-way ANOVA followed by Tukey’s multiple comparison tests.

## Data Availability

Data sharing is not applicable to this article as no datasets were generated or analysed during the current study.

## Abbreviations

AMD: Age-related macular degeneration
ANOVA: Analysis of variance
CNV: Choroidal neovascularization
Col-I: Collagen type I
DAPI: 4′,6-Diamidino-2-phenylindole
DMEM: Dulbecco's modified Eagle medium
ECM: Extracellular matrix
EMT: Epithelial–to-mesenchymal transition
FCS: Foetal calf serum
FFA: Fundus fluorescein angiography
FN: Fibronectin
HRP: Horseradish peroxidase
MMT: Macrophage-to-myofibroblast transition
nAMD: Neovascular age-related macular degeneration
PBS: Phosphate-buffered saline
RIPA: Radioimmunoprecipitation assay buffer
RPE: Retinal pigment epithelia
VEGF: Vascular endothelial growth factor
α-SMA: α-Smooth muscle actin
TGF-β: Transforming growth factor beta
