# Supplementary material for: Complement activation contributes to subretinal fibrosis through the induction of epithelial-to-mesenchymal transition (EMT) in retinal pigment epithelial cells
Source: J Neuroinflammation. 2022 Jul 14;19:182. doi: 10.1186/s12974-022-02546-3 (PMC9447479; doi:10.1186/s12974-022-02546-3)
Supplement: Supplementary file 1 — Additional file 1: Figure S1. The effect of C5a on the expression of fibronectin (FN) and E-cadherin (E-cad) in RPE cells. Primary mouse RPE cells were treated with C5a (50 ng/mL) for different times (A–C) or with different concentrations for 96 h (D-F) and collected for Western blot. (A) Representative Western blot images. (B, C) Quantification of FN (B) and E-cad (D) expression on murine RPE treated with or without C5a for different times. (D) Representative Western blot images from RPE cells treated with different concentrations of C5a (10–100 ng/mL) for 96 h. (E, F) Quantification of FN (E) and E-cad (F) expression on RPE treated with different concentrations of C5a for 96 h. Means ± SEM, n = 3–6 from 2 independent experiments. *p < 0.05 compared to control (0 h); one-way ANOVA followed by Dunnett post hoc test. (G) Representative phase-contrast images of pRPE cells from control, C5a (96 h) and TGF-β2 (96 h) treated group. Scale bare = 50 µm. Figure S2. Effect of C3a on RPE cell epithelial-to-mesenchymal transition. (A, B) Primary murine RPE cells were treated with different concentrations (10, 50, 100 ng/mL) of C3a for 96 h. (C) E-cadherin expression was determined after 96 h treatment with 10 ng/mL of C3a by Western Blot. Mean ± SEM, n = 3–6. (D) Changes in contractility were evaluated in RPE cells after 48 h of C3a (10 and 50 ng/mL) treatment. TGF-β2 (10 ng/mL) was used as a positive control. The area of collagen gel in each group was measured and results were expressed as % of reduction in gel area. Means ± SEM, n = 3–5 gels from 2 independent experiments. ****p < 0.001; one-way ANOVA followed by Dunnett post hoc test compared with untreated. Figure S3. The effect of concomitant treatment of primary mouse RPE (pRPE) cells with C5a and C3a. Murine pRPE cells were treated for 96 h with C3a (10 ng/mL) and C5a (50 n/mL). The expression of mesenchymal markers α-SMA and FN, along with epithelial marker E-cadherin was examined by Western Blot. n = 2–4. *p < 0.05; * [file 12974_2022_2546_MOESM1_ESM.docx]

**Additional file**

**

**

**Supplementary Figure S1**. The effect of C5a on the expression of fibronectin (FN) and E-cadherin (E-cad) in RPE cells. Primary mouse RPE cells were treated with C5a (50 ng/mL) for different times (A-C) or with different concentrations for 96 h (D-F) and collected for Western blot. (A) Representative Western blot images. (B, C) Quantification of FN (B) and E-cad (D) expression on murine RPE treated with or without C5a for different times. (D) Representative Western blot images from RPE cells treated with different concentrations of C5a (10-100 ng/mL) for 96 h. (E, F) Quantification of FN (E) and E-cad (F) expression on RPE treated with different concentrations of C5a for 96 h. Means ± SEM, n = 3-6 from 2 independent experiments. * p< 0.05 compared to control (0 h); One way ANOVA followed by Dunnet post-hoc test. (G) Representative phase-contrast images of pRPE cells from control, C5a (96h) and TGF-β2 (96h) treated group. Scale bare = 50 µm.


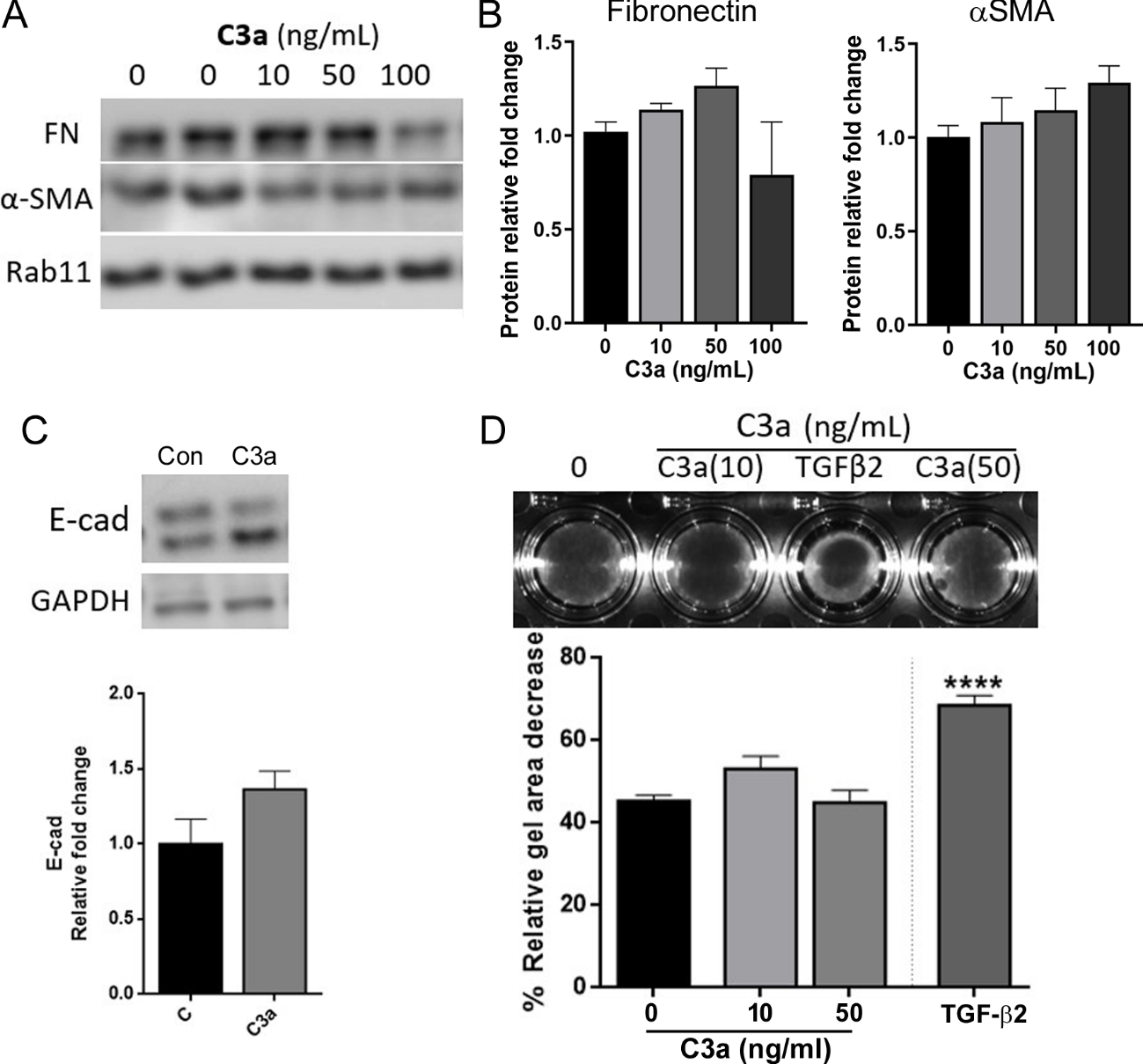


**Supplementary Figure S2.** Effect of C3a on RPE cell epithelial-to-mesenchymal transition. (A, B) Primary murine RPE cells were treated with different concentrations (10, 50, 100ng/mL) of C3a for 96 h. (C) E-cadherin expression was determined after 96 h treatment with 10ng/mL of C3a by Western Blot. Mean ± SEM, n=3-6. (D) Changes in contractility were evaluated in RPE cells after 48 h of C3a (10 and 50ng/mL) treatment. TGF-β2 (10ng/mL) was used as a positive control. The area of collagen gel in each group was measured and results were expressed as % of reduction in gel area. Means ± SEM, n**=**3-5 gels from 2 independent experiments. **** p< 0.001; One way ANOVA followed by Dunnet post-hoc test compared with untreated.


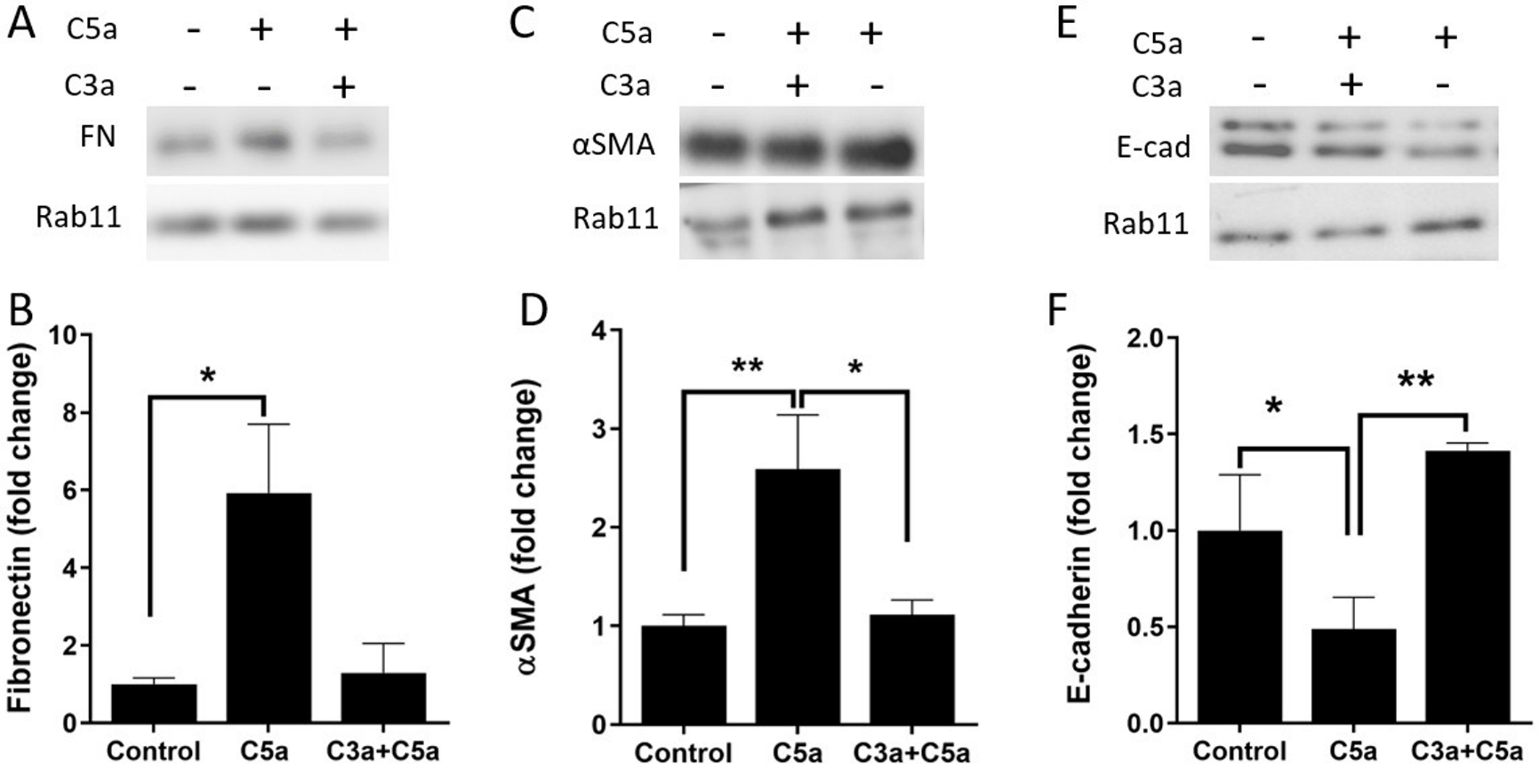


**Supplementary Figure S3.** The effect of concomitant treatment of primary mouse RPE (pRPE) cells with C5a and C3a. Murine pRPE cells were treated for 96h with C3a (10ng/mL) and C5a (50 n/mL). The expression of mesenchymal markers αSMA and FN, along with epithelial marker E-cadherin was examined by Western Blot. n = 2-4. * p< 0.05; ** p< 0.01, One way ANOVA followed by Tukey’s multiple comparison tests.
